# SynGenes: a Python class for standardizing nomenclatures of mitochondrial and chloroplast genes and a web form for enhancing searches for evolutionary analyses

**DOI:** 10.1186/s12859-024-05781-y

**Published:** 2024-04-22

**Authors:** Luan Pinto Rabelo, Davidson Sodré, Rodrigo Petry Corrêa de Sousa, Luciana Watanabe, Grazielle Gomes, Iracilda Sampaio, Marcelo Vallinoto

**Affiliations:** 1 Laboratório de Evolução, IECOS, Universidade Federal do Pará, Campus de Bragança, Bragança, Brazil; 2 Universidade Federal Rural da Amazônia (UFRA), Campus de Capitão Poço, Capitão Poço, Brazil; 3 Laboratório de Genética Aplicada (LAGA), IECOS, Universidade Federal do Pará, Campus de Bragança, Bragança, Brazil; 4 CIBIO-InBIO, Centro de Investigação em Biodiversidade e Recursos Genéticos, Universidade do Porto, Porto, Portugal

**Keywords:** Genomic data, Genbank, Bioinformatics, Synonymous names

## Abstract

**Background:**

The reconstruction of the evolutionary history of organisms has been greatly influenced by the advent of molecular techniques, leading to a significant increase in studies utilizing genomic data from different species. However, the lack of standardization in gene nomenclature poses a challenge in database searches and evolutionary analyses, impacting the accuracy of results obtained.

**Results:**

To address this issue, a Python class for standardizing gene nomenclatures, SynGenes, has been developed. It automatically recognizes and converts different nomenclature variations into a standardized form, facilitating comprehensive and accurate searches. Additionally, SynGenes offers a web form for individual searches using different names associated with the same gene. The SynGenes database contains a total of 545 gene name variations for mitochondrial and 2485 for chloroplasts genes, providing a valuable resource for researchers.

**Conclusions:**

The SynGenes platform offers a solution for standardizing gene nomenclatures of mitochondrial and chloroplast genes and providing a standardized search solution for specific markers in GenBank. Evaluation of SynGenes effectiveness through research conducted on GenBank and PubMedCentral demonstrated its ability to yield a greater number of outcomes compared to conventional searches, ensuring more comprehensive and accurate results. This tool is crucial for accurate database searches, and consequently, evolutionary analyses, addressing the challenges posed by non-standardized gene nomenclature.

**Graphical abstract:**

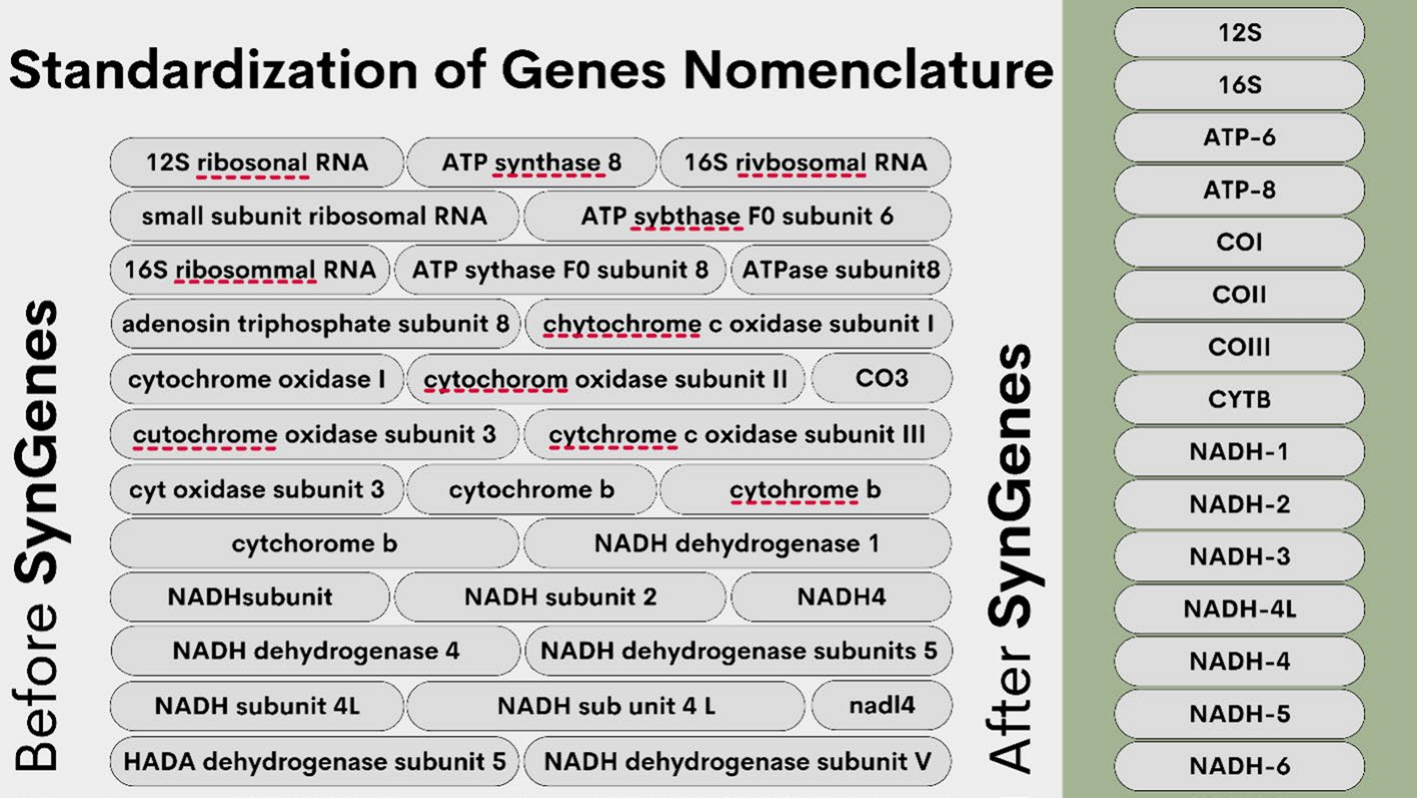

**Supplementary Information:**

The online version contains supplementary material available at 10.1186/s12859-024-05781-y.

## Background

The reconstruction of the evolutionary history of various organisms has been greatly influenced by the advent of molecular techniques [1, 2], which are becoming increasingly faster and more accessible, enabling the acquisition of data of higher quality and quantity. Consequently, a large volume of genomic data has been produced for evolutionary studies of diverse organisms [3–7]. These advancements have led to a significant increase in studies and scientific publications utilizing genomic data from different species.

The search for genes or genomes to be used in evolutionary studies often faces the challenge of lack of standardization in nomenclature, which can significantly impact the results obtained during searches. For example, when conducting a search on the National Center for Biotechnology Information (NCBI)—GenBank or on PubMedCentral for a specific gene or for relevant studies that utilize a certain gene, it is common to use the species name (e.g. the white sharks, *Carcharodon Carcharias*, or the blacktip sharks, *Carcharhinus limbatus*) along with the desired gene abbreviation (*COI*). However, this type of search may yield a reduced number of results compared to advanced searches that utilize the species name together with keyword combinations, such as alternative gene names and synonyms (e.g., (“*Carcharodon Carcharias*”[Organism]) AND (“COI”[All Fields] OR “cytochrome oxidase subunit I”[All Fields] OR “cytochrome c oxidase subunit 1”[All Fields])) [8].

To utilize this type of advanced search, it is necessary to incorporate gene name variations, and researchers need to have a database containing such synonyms to construct their search and obtain more accurate results. Therefore, in the field of conservation studies [9], taxonomic revisions [10], creation of databases for metabarcoding references [11, 12], and species identification [13–15], obtaining comprehensive results from databases is of utmost importance to conduct thorough and precise analyses.

**Fig. 1:**
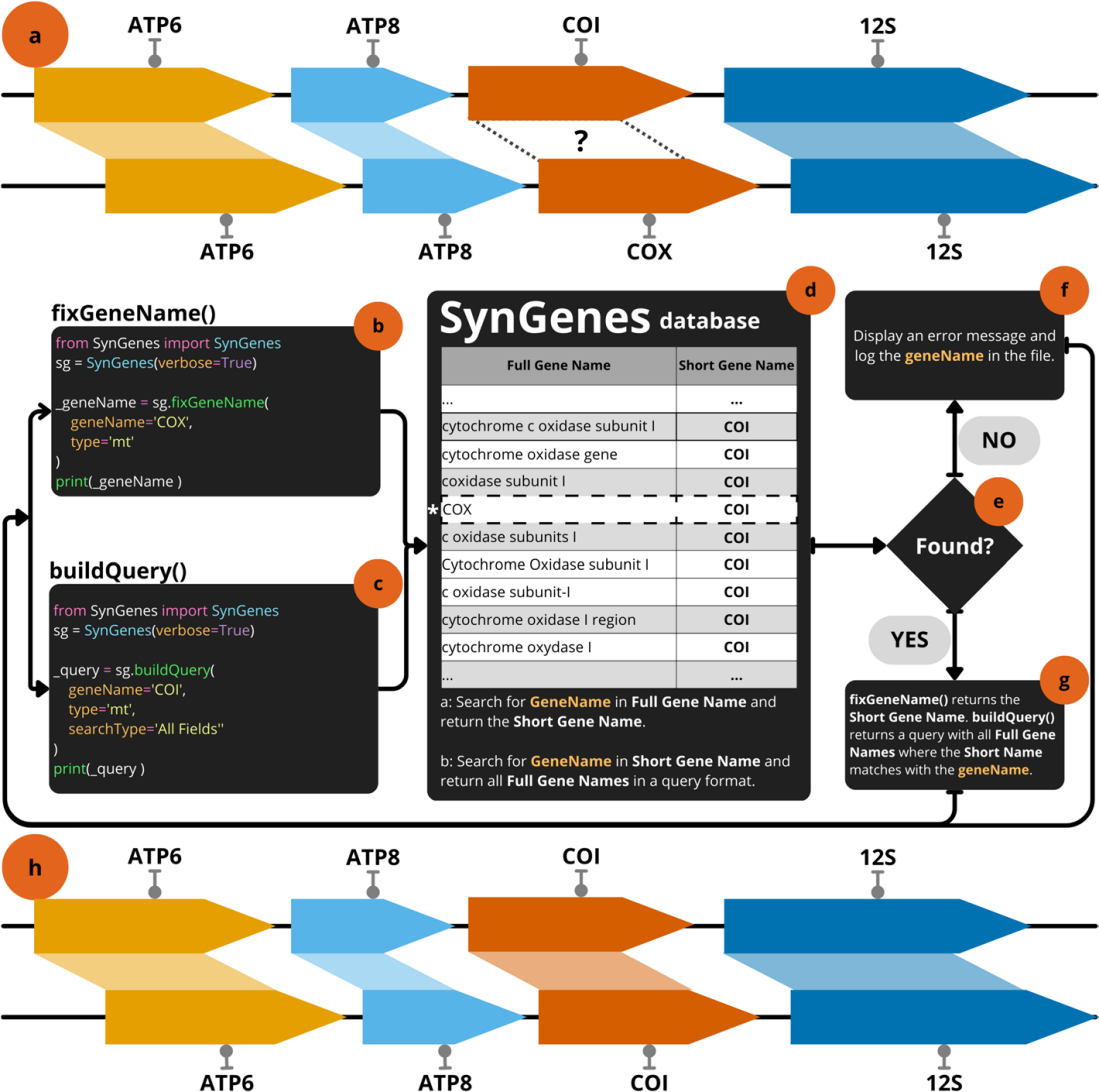
The lack of standardization in gene nomenclature can lead to inconsistencies in analyses (**a**). SynGenes Python Class possess two main functions, gene name standardization (**b**), and query construction (**c**). The first function requires the user to input the long gene name and the type (mt for mitochondrial genes or cp for chloroplast genes), as mandatory parameters. Upon execution of the standardization function, the provided name is searched in the database (**d**), returning the corresponding short name if it exists (**e**, **f**). If the information is not found (**e**), an error message is displayed, and the unfound name is logged into a file (**g**). This process helps in resolving such inconsistencies for posterior genetic analyses (**h**)

A lack of standardization in gene nomenclature poses a challenge not only in database searches like NCBI or PubMedCentral, but also in more complex evolutionary analyses, such as rearrangements of gene orders in mitochondrial and chloroplast genomes [16, 17]. In such cases, it is crucial for gene names to be consistent to enable accurate comparisons (Fig. 1a). For instance, in the context, "cytochrome oxidase subunit I" gene could be recognized as different from "cytochrome c oxidase subunit 1," which can lead to misinterpretation of data by tools like CREx [18], qMGR [19] or PhyloSuite [20]. To ensure proper comparison of these genes by analysis tools, it is necessary to standardize them using a common abbreviation, such as “*COI*”. Tools that rely on standardized nomenclature, to perform a proper analysis, can generate confusing results if they do not use appropriate standardization (Fig. 1a). Therefore, non-uniform gene names not only hinder gene searches in databases, like GenBank, but also complicate the automatic preparation of input files for analyses reliant on this information.

One tool that can be used to obtain synonymous of gene names is the BioKDE [21] platform (https://www.biokde.com/). This is a powerful tool that harnesses deep learning algorithms to analyze vast amounts of scientific literature from PubMedCentral, extracting valuable information from this data. One of its key features is the ability to identify and visualize relationships between genes, proteins, and diseases, providing valuable insights for researchers and practitioners. Additionally, BioKDE boasts a comprehensive database of gene synonyms sourced from the analyzed scientific manuscripts, enhancing its utility for users. However, it is important to note that while BioKDE is an online platform with robust capabilities, it currently lacks an API [21], limiting its integration into standardized workflows. Also, it is worth mentioning that previous analyses (data not shown) reveal that the number of gene synonyms obtained by BioKDE is still limited.

To address the issue of inconsistent gene names in mitochondrial and chloroplast genomes, we present SynGenes (**Syn**onyms of **Genes**) (Fig. 1b–g), a repository freely available on GitHub (https://github.com/luanrabelo/SynGenes) under the MIT license, which provides standardization of gene names using a consistent nomenclature. The SynGenes repository is an open-source resource that provides researchers with the results of the analysis of mitochondrial and chloroplast gene names, along with detailed instructions and example scripts.

Additionally, we have created a user-friendly web form to assist researchers in performing specific gene, genome or manuscripts searches in GenBank or PubMedCentral using different gene nomenclatures. This tool will enable researchers to utilize the various existing gene or genome nomenclatures (Mitochondrial and/or Chloroplast), ensuring a more comprehensive and accurate search.

### Implementation

The SynGenes Class is designed to be implemented in various workflows and requires a functional Python 3.10 + environment and an internet connection. SynGenes can be easily installed via PyPI using the command "pip install SynGenes". Comprehensive documentation on how to utilize the class is available on the PyPI project page (https://pypi.org/project/SynGenes/), as well as in the GitHub repository (https://github.com/luanrabelo/SynGenes).

To utilize SynGenes, it is necessary to have some open-source packages installed, including requests (version > 2.30.0) and pandas (version > 2.0.1). However, it is worth noting that the script already includes modules for installing these packages in the case they are not previously installed on the user's computer. This ensures that the necessary packages are readily available for their use with SynGenes.

The SynGenes class only requires an internet connection for package installation and to obtain the database for the user's computer. This process can be done during the initial execution of the script or when the user requests an update to the database (check the documentation of SynGenes at Github for instructions).

In addition to the repository, we have developed a web form (https://luanrabelo.github.io/SynGenes/) to accommodate researchers who wish to perform individual searches using different names associated with the same gene. This web form generates a command that incorporates multiple gene names, enabling precise searches on the GenBank or PubMedCentral platform.

### SynGenes functions and database

SynGenes Python Class possess two main functions. The first one is related to the gene name standardization (Fig. 1b), and query construction (Fig. 1c). The first function requires the users to input the long or short gene names and the type of organelle. The second function allows users to make a search query with any gene name variants for automatic searches in databases. When the standardization function is executed, the provided name is searched in the database (Fig. 1d; see below), returning the corresponding short name if it exists (Fig. 1e, g) or an error (Fig. 1f).

To build a database of synonymous gene names, data obtained from the analysis of gene names and gene products of mitochondrial and chloroplast genomes of Chordata and Embryophyta were utilized. The Entrez and SeqIO modules of Biopython were used for genome retrieval and reading, while Pandas was employed for data manipulation. The query used to search for Chordata genomes was ("Chordata"[Organism] OR "Chordata"[All Fields]) AND ("Genome"[Title]) AND ("mitochondrial"[Title] OR "mitochondrion"[Title]) AND mitochondrion[filter]. While for Embryophyta, the query was ("Embryophyta"[Organism] OR "Embryophyta"[All Fields]) AND ("Genome"[Title]) AND chloroplast[filter].

We decided, for the database construction, to analyze the names of genes contained in different genomes, rather than obtaining the maximum number of different names for each specific gene. The motive relies on the fact that, when analyzing a single genome, it is possible to obtain (at most) only 37 different nomenclatures for each mitochondrial gene and about 120 nomenclatures for each chloroplast genes. Therefore, using thousands of genomes we can obtain easily different names adopted for each marker. In addition, obtaining thousands of individual genes for analysis would require high processing power and storage capacity. The analysis of only the genomes of Chordates and Embryophytes resulted in 5 and 10 GB of disk storage, respectively. In May 2023, a total of 99,203 mitochondrial genomes and 69,087 chloroplast genomes were obtained for analysis (see Additional files 1 and 2).

**Fig. 2:**
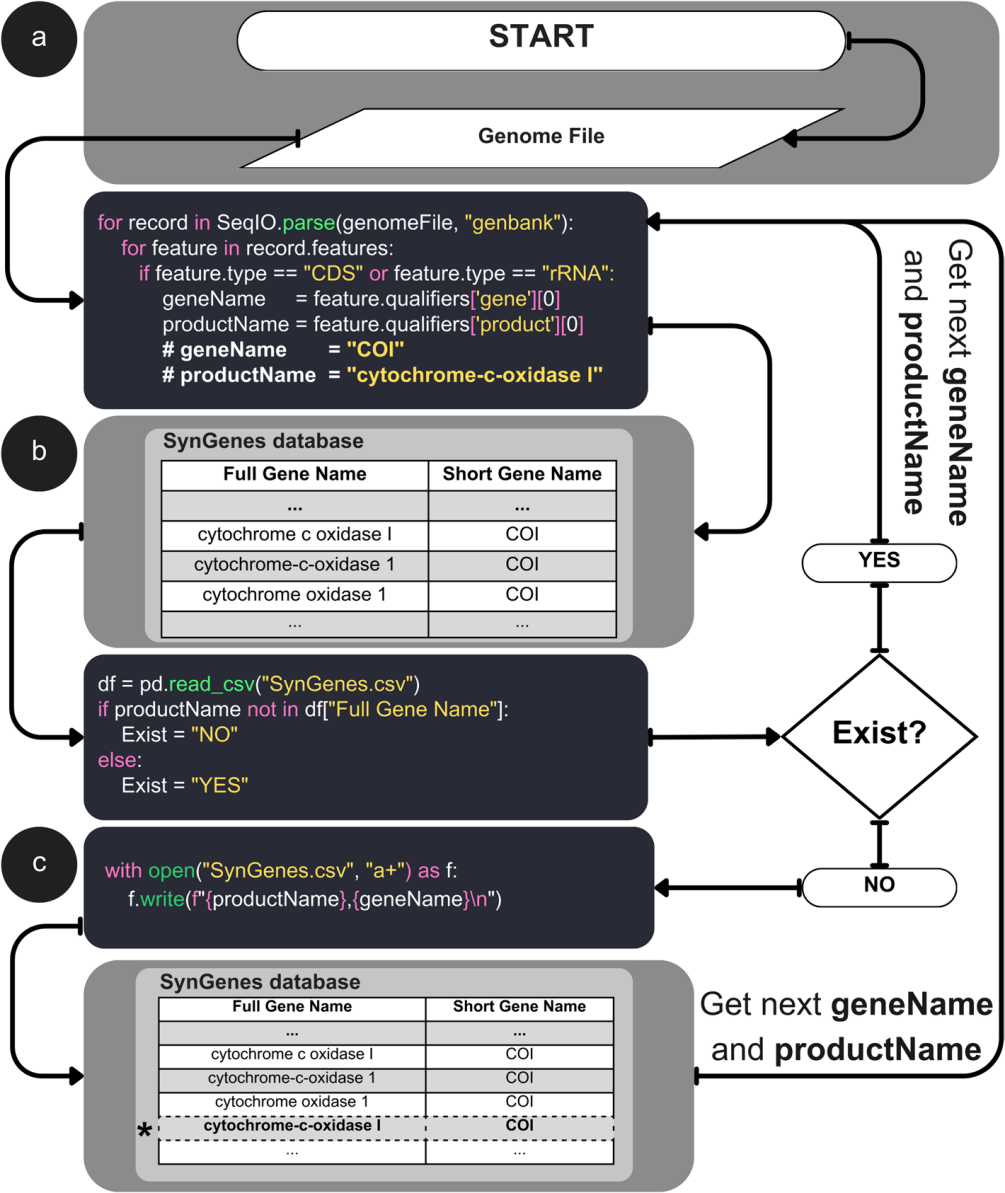
Workflow for building the SynGenes database. **a** Parse each genome file to extract gene and product names for coding and ribosomal genes. **b** Check if the product name exists in the SynGenes database using panda’s library. **c** If the product name does not exist, write it into the database along with the gene name. The process repeats until all the genome files are processed

The process of constructing the SynGenes database is illustrated in Fig. 2. In this process, the first stage (Fig. 2a) involves analyzing each genome to extract the gene product name and the associated gene name. This analysis is specifically conducted for coding genes (CDS) and ribosomal genes (rRNA). Subsequently, in the second stage (Fig. 2b), a database query is performed to check if the gene product name is already registered. This query is executed using the panda’s library with the *read_csv* method. If the gene product name already exists in the database, the process returns to stage (a) and continues with the next iteration, obtaining the names of the next gene product and gene. If the gene product name does not exist in the database, it is inserted along with the corresponding gene name (Fig. 2c). After the insertion, the process returns to the first stage (a) to obtain the next gene product name. This methodology ensures the generation of a list of gene product names without redundancy, as each entry is verified before insertion.

We also collected the variations of the gene names on the bioKDE platform, where a manual search for each gene (mitochondrial and chloroplast) were conducted and the results were included in the SynGenes database.

### Standardizing gene names with HUGO

After obtaining the structured databases, the coding and ribosomal genes commonly used in phylogenetics were identified and added to their nomenclature based on rules established by the Human Genome Organization (HUGO) [22]. For example, the gene "*COX1*," found in some mitochondrial genomes, were renamed to "*COI*." Table 1 shows the gene names established by HUGO.

**Table 1:** List of the names of the mitochondrial and chloroplast genes standardized according to the HUGO nomenclature

Mitochondrial genes		Chloroplast genes	
rRNA	*12S; 16S*	rRNA	*rrn16S; rrn23S; rrn4.5S; rrn5S*
Mitochondrial Complex I	*ND1; ND2; ND3; ND4; ND4L; ND5; ND6*	ATP Synthase	*atpA; atpB; atpE; atpF; atpH; atpI*
Mitochondrial Complex III	*CYTB*	Cytochrome b/6f Complex	*petA; petB; petD; petE; petG; petL; petN*
Mitochondrial Complex IV	*COI; COII; COIII*	DNA dependent RNA polymerase	*rpoA; rpoB; rpoC1; rpoC2*
Mitochondrial Complex V	*ATP6; ATP8*	Large Subunit of Ribosome	*rpl2; rpl14; rpl16; rpl20; rpl22; rpl23; rpl32; rpl33; rpl36*
Control Region	Control Region	NADH-dehydrogenase	*ndhA; ndhB; ndhC; ndhD; ndhE; ndhF; ndhG; ndhH; ndhI; ndhJ; ndhK*
		PhotoSystem I-II	*psaA; psaB; psaC; psaI; psaJ; psaM; psb30; psbA; psbB; psbC; psbD; psbE; psbF; psbH; psbI; psbJ; psbK; psbL; psbM; psbN; psbZ*
		Small Subunit of Ribosome	*rps2; rps3; rps4; rps7; rps8; rps11; rps12; rps14; rps15; rps16; rps18; rps19*
		Rubisco	rbcL
		Others	*accD; ccsA; cemA; chlB; chlL; chlN; clpP; clpP1; cysA; cysT; ftsH; infA; lhbA; matK; pafI; pafII; pbf1; psb30; ycf1; ycf2; ycf3; ycf4; ycf12; ycf15;*

## Results

**Fig. 3:**
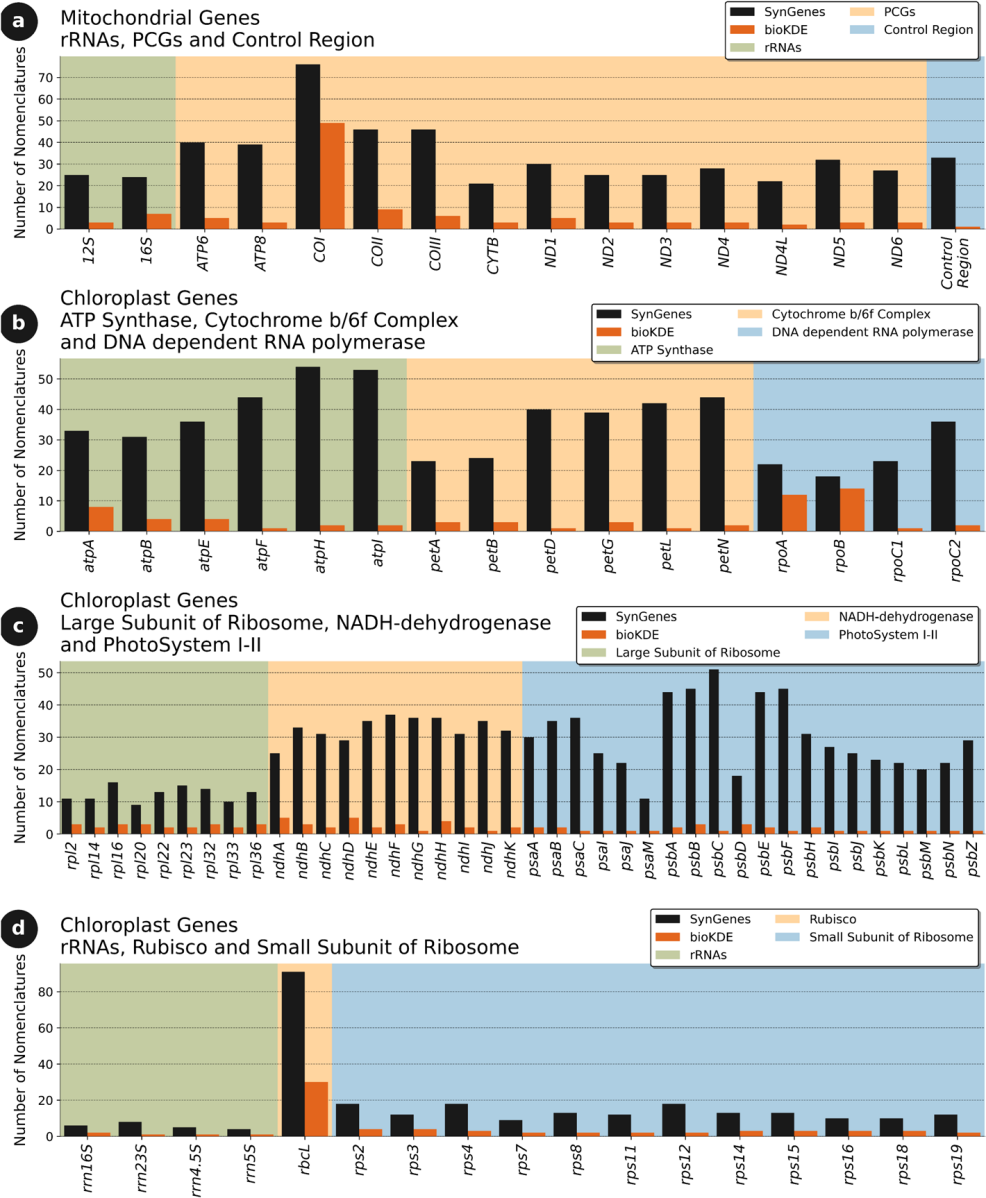
Analysis of the number of synonyms of mitochondrial gene (**a**) and chloroplast gene names (**b**–**d**) present in the SynGenes database (v1.0.1) in relation to the bioKDE database

The SynGenes database (v1.0.1) contains a total of 545 gene name variations for mitochondrial and 2485 for chloroplasts (list of all gene variations may be found in GitHub repository). A comparison with other synonym databases available in the literature, such as bioKDE [21], is illustrated in Fig. 3.

In terms of its Python class, it comprises five functions detailed in Table 2. These functions facilitate the updating of the SynGenes database on the user's computer and enable rapid standardization, if necessary. This is particularly useful for gene names present in mitochondrial and chloroplasts genomes, ensuring their compatibility with automated tools for creating input files that require standardized names [18–20]. Furthermore, it includes a function for generating commands that can be used in advanced searches of genes (GenBank) and manuscripts (PubMedCentral) using the Entrez tool. Lastly, there is a function that exports the SynGenes database to the JSON format, allowing its implementation in tools that utilize other programming languages, such as NodeJS. It is worth noting that the SynGenes repository contains several example Python files for utilizing all these functions.

**Table 2:** List of functions of the SynGenes class in Python

Function name	Function	Parameters
updateSynGenes()	Download SynGenes database from GitHub repository (stable branch)	**verbose (bool)**: Print messages. Default is True
fixGeneName()	Fix Gene Name according to the SynGenes database	**geneName (str)**: Gene name to be fixed **type (str)**: Type of gene (mt = Mitochondrial, cp = Chloroplast). Default is mt **verbose (bool)**: Print messages. Default is True
buildQuery()	Build a query for Entrez search	**geneName (str)**: Gene name to search, must be in the correct format, use the function **fixGeneName()** to fix the gene name **type (str)**: Type of gene (mt = Mitochondrial, cp = Chloroplast). Default is mt **searchType (str)**: Type of search (Title, Abstract, All Fields, MeSH Terms). Default is All Fields **verbose (bool)**: Print messages. Default is True
buildJson()	Build a JSON file with the data of SynGenes database	**fileName (str)**: Name of the JSON file. Default is SynGenes.js **pathSaveFile (str)**: Path to save the JSON file. Default is SynGenes folder, in the current working directory **verbose (bool)**: Print messages. Default is True
versionSynGenes()	Show the version of SynGenes database	**None**

### Computation time

The Python class we have developed demonstrates the capability to analyze 1000 genomes of diverse origins, encompassing both mitochondrial and chloroplast genomes, within a short timeframe. To assess computational time, we utilized a computer equipped with an AMD Ryzen 7 5000 Series processor and 16 GB of RAM. Additionally, we simulated the creation of a text file containing the names of protein-coding genes (PCGs) and ribosomal RNAs (rRNAs) present in the genomes. We randomly selected 1000 genomes available for consultation (see Additional files 1 and 2) and conducted the data analysis.

**Fig. 4:**
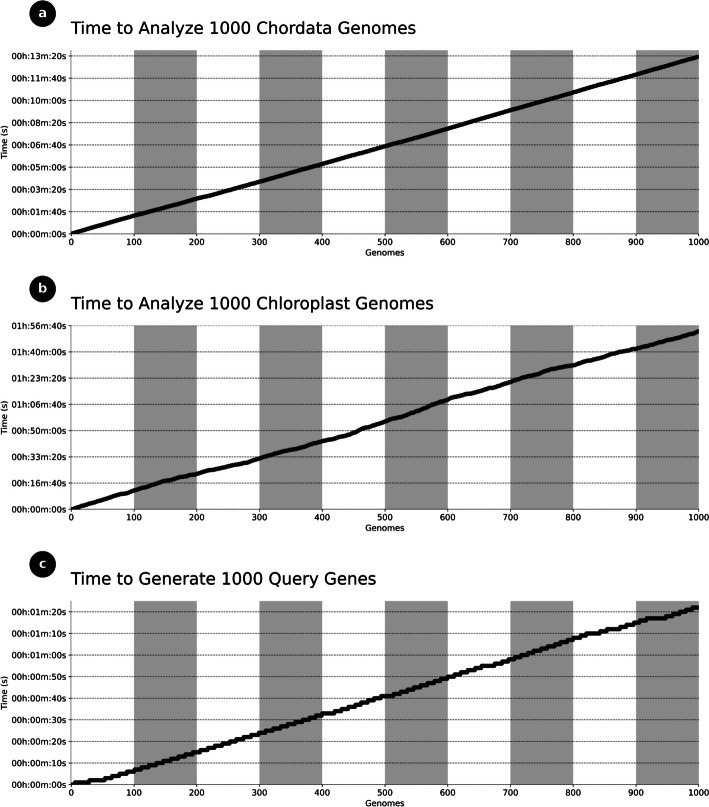
**a** Time required to standardize the names of the genes that encode proteins in the mitochondrial genomes. **b** Time required to standardize the names of the genes that encode proteins in the chloroplast genomes. **c** Average time to generate 1000 queries for advanced searches, using the synonyms, to be used in tools like Entrez

The results revealed that our Python class efficiently processed the data in less than 14 min for the chordate genomes (Fig. 4a) and approximately 2 h for chloroplast genomes (Fig. 4b). Furthermore, our Python class generates commands that can be used for advanced and automated searches using the Entrez tool, an integrated search system for various biological databases, or by utilizing the forms present on the GenBank and PubMedCentral websites. The time required to generate the 1000 commands was less than 90 s (Fig. 4c).

### Search data in Genbank and PubMedCentral

**Fig. 5:**
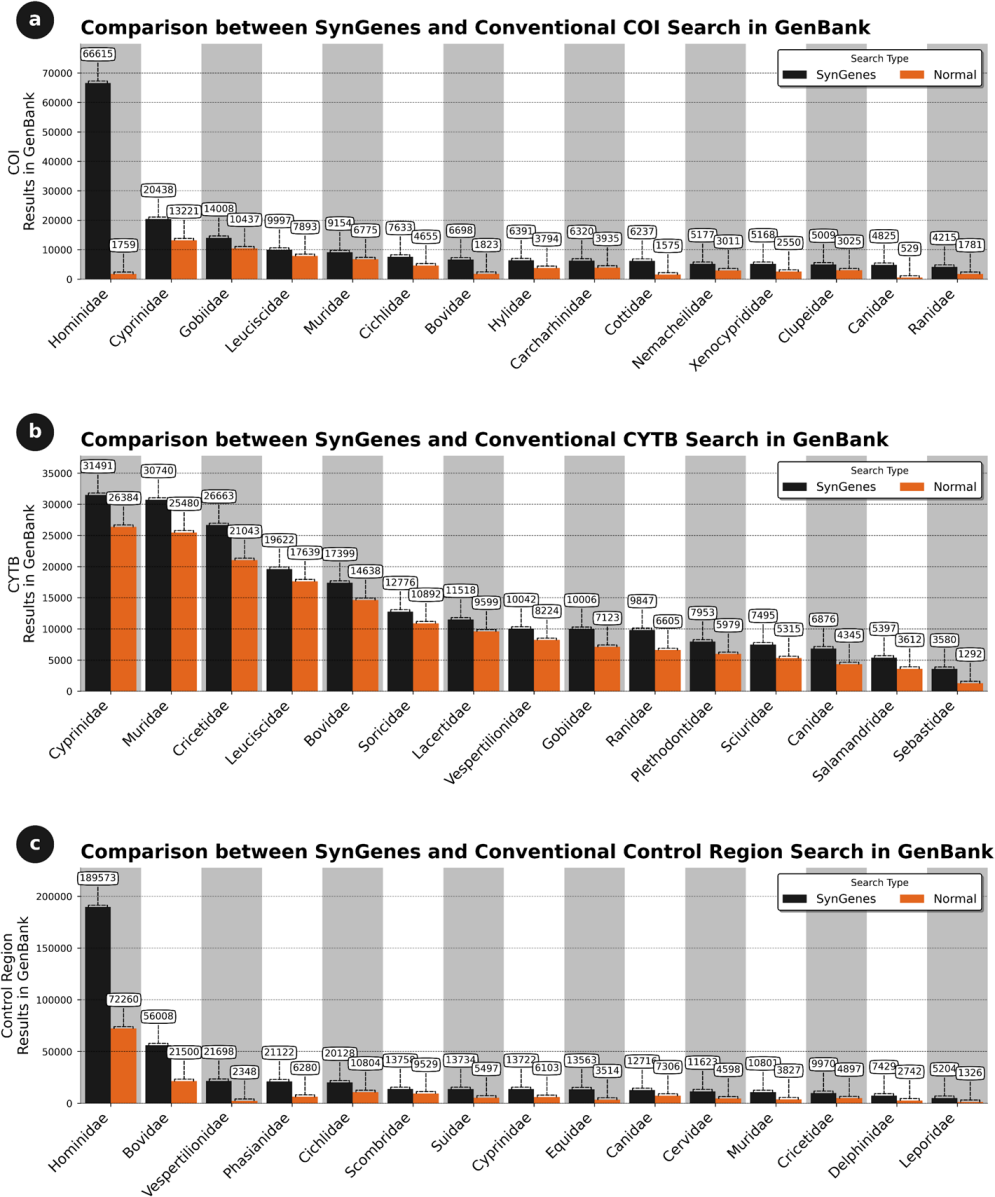
Comparison of search results obtained in January 2024 from the SynGenes web form versus traditional searches in GenBank, highlighting the higher efficiency achieved by using combinations of gene nomenclature for *COI* (**a**), *CYTB* (**b**) and Control Region markers (**c**)

The effectiveness of SynGenes was evaluated through research conducted on GenBank and PubMedCentral, focusing on the *COI*, *CYTB* genes and the *Control Region*, which are frequently employed in phylogenetic and population studies [14, 23–26]. The investigation encompassed 944 chordate families (Additional file 1). The results indicated that SynGenes obtained a greater number of outcomes by utilizing gene name variations compared to traditional searches in GenBank using only family and gene names. Specifically, for the *COI* gene, SynGenes yielded more results for 94.17% of the analyzed chordate families compared to traditional searches. That is, in all comparisons between SynGenes and traditional search methods, the former outperformed in approximately 94% all searches. In some instances, SynGenes showed slight improvement, while in others, the difference was significantly higher. For example, in the case of Hominidae, the traditional search yielded 1,759 results, whereas with SynGenes, it produced 66,515 results (Fig. 5). This highlights the substantial advantage of using SynGenes over conventional search methods.

For the *CYTB* gene, this occurred for 99.15% of the families, and for the *Control Region*, for 88.98% of the families (data not shown). Figure 5 presents only 20 results between SynGenes versus traditional searches from different chordate families of this comprehensive analysis. Those comparisons highlight the results where the difference between SynGenes versus traditional searches demonstrate the best performance of the former.

**Fig. 6:**
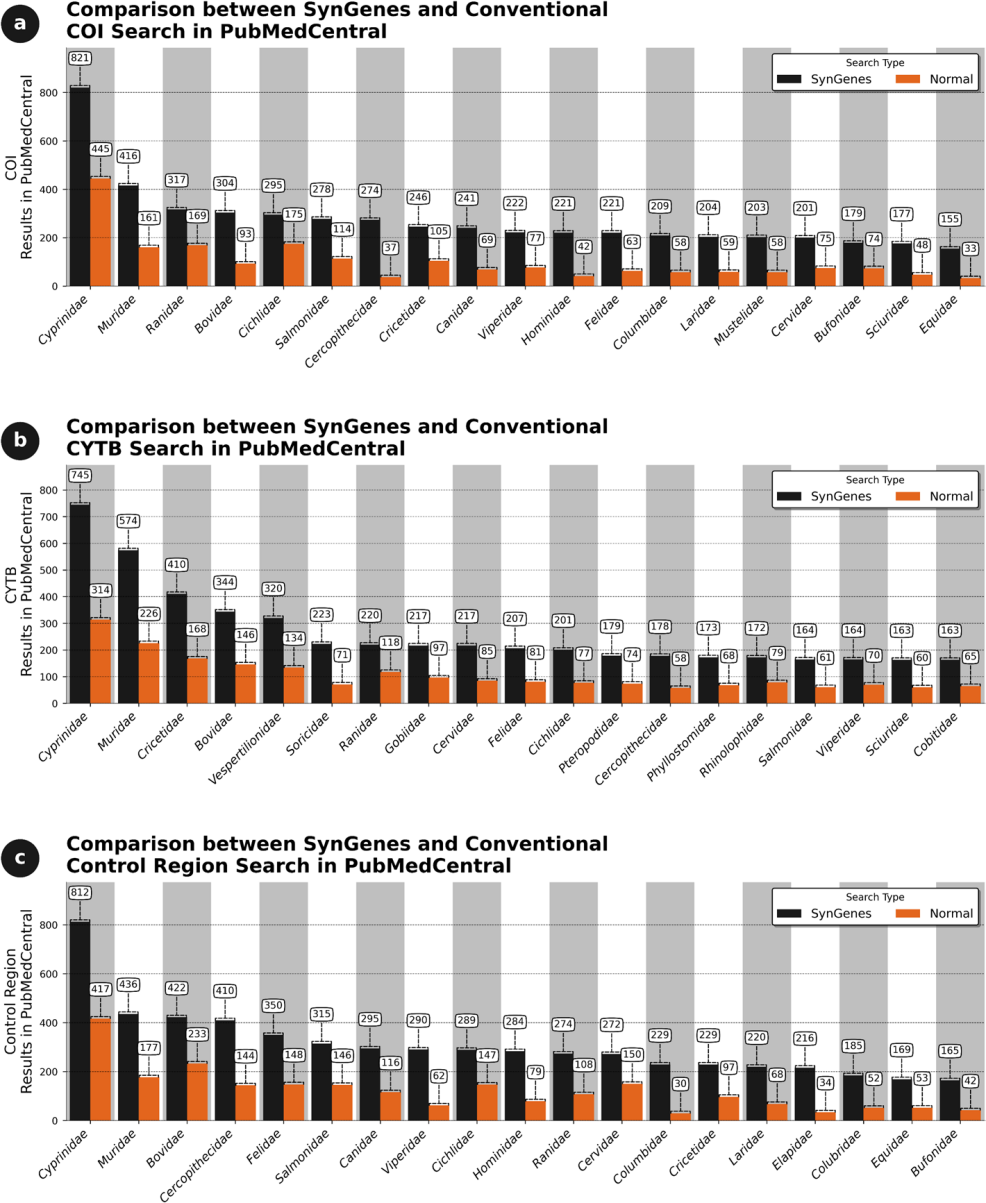
Comparison of the efficiency of the searches performed in January 2024 by the SynGenes web form and the traditional searches on PubMedCentral, showing the benefit of using combinations of gene nomenclature for *COI* (**a**), *CYTB* (**b**) and Control Region markers (**c**)

In PubMedCentral, using the same families and gene combinations, an 82.94% increase for *COI* was achieved compared to traditional searches. For *CYTB*, there was a 78.70%, and for the *Control Region*, an 88.24% (data not shown). Figure 6 displays 20 results from different chordate families obtained from the best performances of SynGenes versus traditional searches.

**Fig. 7:**
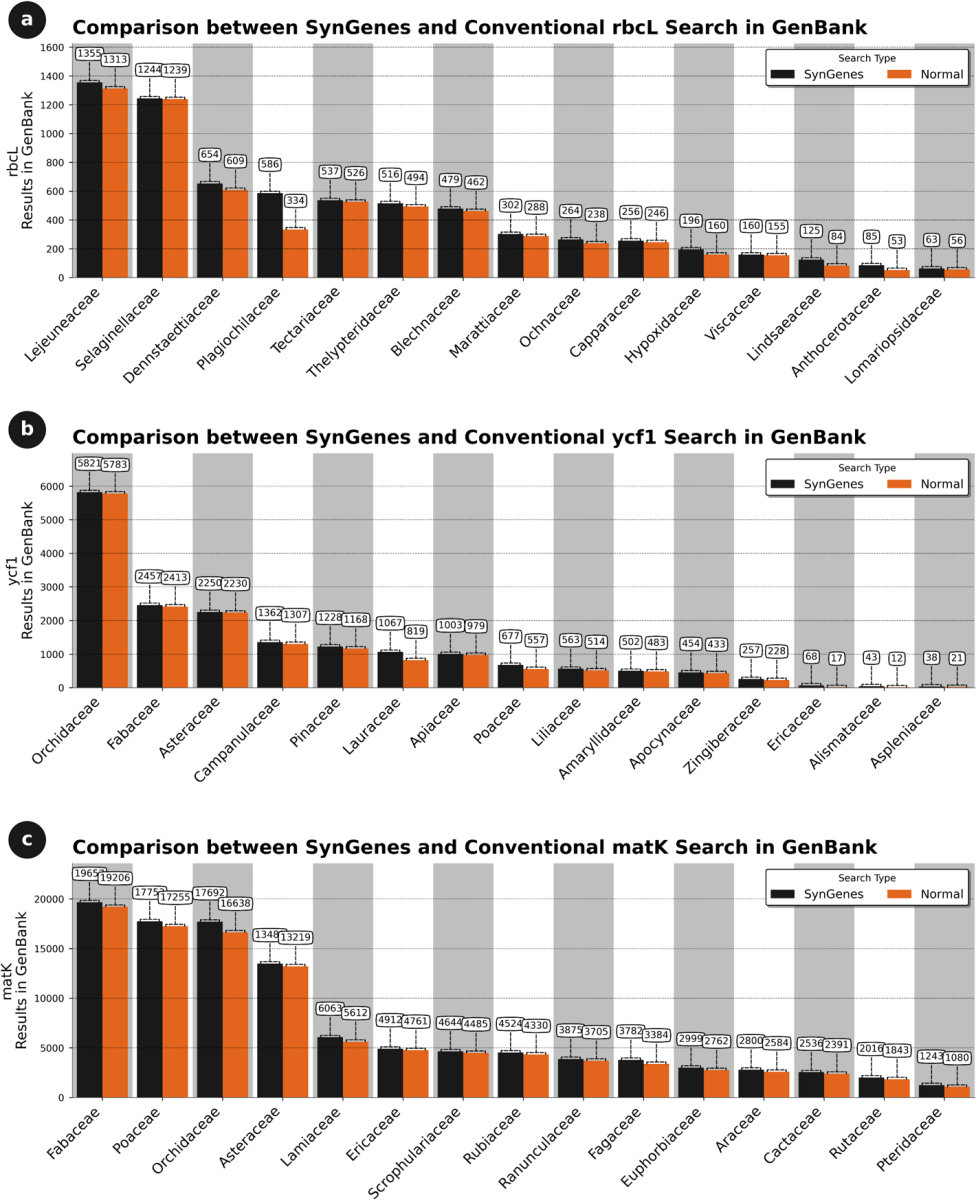
Comparison of search results obtained in January 2024 from the SynGenes web form versus traditional searches in GenBank, highlighting the higher efficiency achieved by using combinations of gene nomenclature for *rbcL* (**a**), *ycf1* (**b**) and *matK* markers (**c**)

**Fig. 8:**
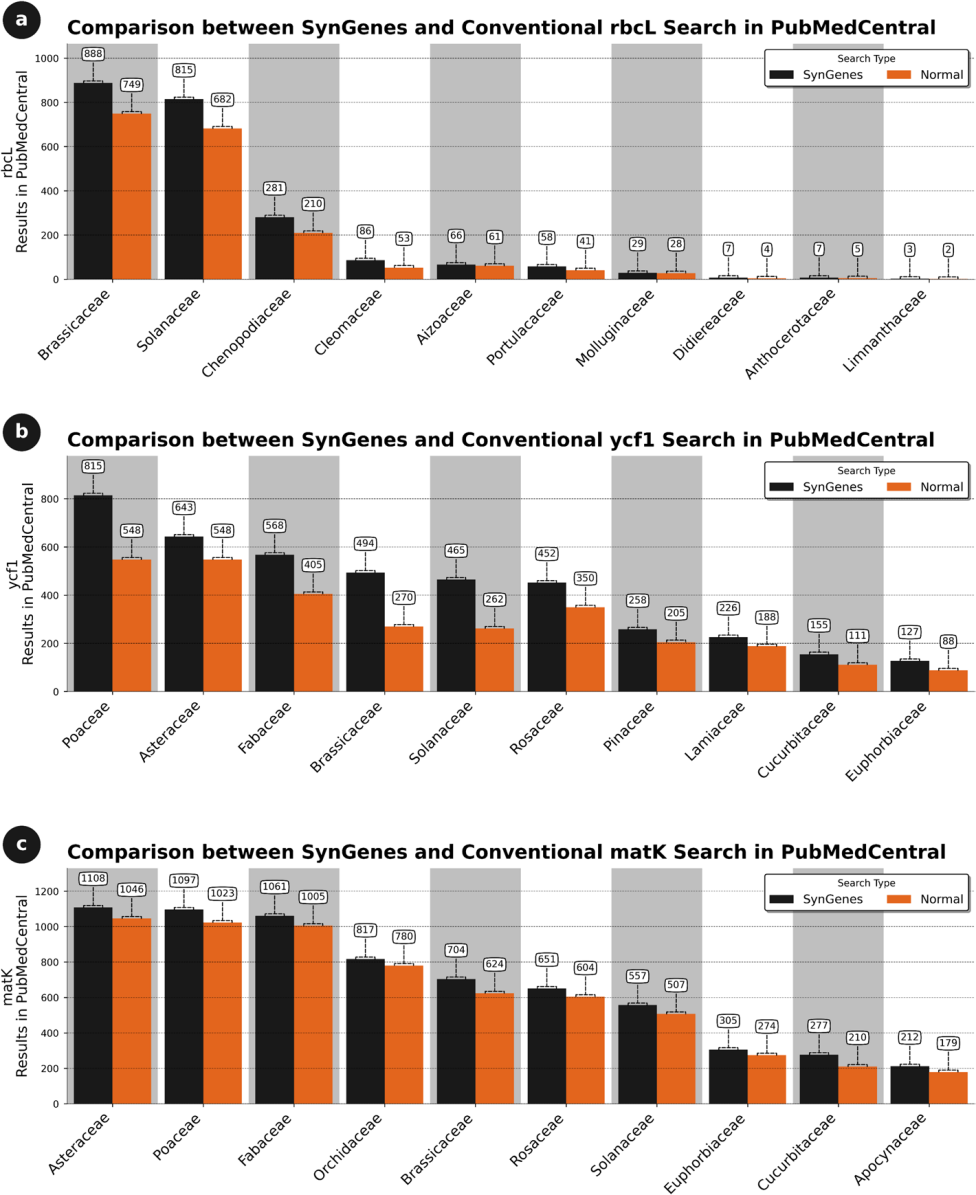
Comparison of the efficiency of the searches performed in January 2024 by the SynGenes web form and the traditional searches on PubMedCentral, showing the benefit of using combinations of gene nomenclature for *rbcL* (**a**), *ycf1* (**b**) and *matK* markers (**c**)

For chloroplast genes, the *rbcL*, *ycf1*, and *matK* genes in 474 terrestrial plant families (embryophytes) were analyzed, as described in Additional file 2. Sequences of these genes were sought in the GenBank and PubMedCentral databases. Comparing the results obtained from both databases, it was observed that SynGenes presented a higher number of results for all three genes compared to traditional GenBank searches, while PubMedCentral exhibited lower coverage. For example, the *rbcL* gene, encoding the large subunit of the Rubisco enzyme, SynGenes had 22.74% more results observed than traditional GenBank searches, while 2.32% more results than in PubMedCentral traditional searches (data not shown). The *ycf1* gene, encoding a chloroplastic gene expression factor, had 88.81% and 46.83% more results than GenBank and PubMedCentral traditional searches, respectively (data not shown). Finally, *matK* gene, encoding a specific intron maturase, SynGenes had 97.89% and 51.47% more results than in GenBank and PubMedCentral traditional searches, respectively (data not shown). Figures 7 and 8 illustrate 15 results for searches in GenBank and PubMedCentral, respectively, obtained from the best performances of SynGenes versus traditional searches.

The searches in GenBank and PubMedCentral were conducted using specific filters for each database, therefore, to avoid biases and obtain more precise and relevant results. The filters were based on criteria’s, such as molecule (Mitochondrial or Chloroplast) and sequence type (Nucleotide). It is worth mentioning that, in some cases, for instance, a single search for a particular species along with the desired gene/maker, in the GenBank nucleotide database, yielded more results than using SynGenes. This is simply explained by the fact that this search added more results, because it contained mitogenomes, genomes, or files from RNA-Seq assemblies. All of this contained the searched terms, however, even so, those results were not necessarily relevant to the research objective.

## Conclusions

Several variations in the nomenclature of the same gene have been identified in mitochondrial and chloroplasts genomes, some of which are the result of human errors, such as "cytchrome" (KY018081.1), "chytochrome" (LC631940.1), and "cutochrome" (AB052080.1) in mitochondrial genomes, and "ATP synthaes", "ATP synthase", or "ATP-synthase" in chloroplasts. In addition, the lack of standardization also contributes to these variations in nomenclature. This lack of uniformity hinders the search and automated analysis of these genes in genomic databases, as well as the correct interpretation of the obtained results.

Non-standardized nomenclatures clearly hinder the search for genes in databases, such as GenBank, and the automated preparation of input files for subsequent analyses that use this information. This can be particularly problematic when using analysis tools such as CREx [18], qMGR [19], or PhyloSuite [20], which rely on standardized nomenclature for proper functioning. Standardized gene nomenclature facilitates posterior genetic/evolutionary analyses and the potential obstacles posed by inconsistent gene naming conventions (Fig. 1h). Tools like SynGenes exemplify efforts to streamline data processing by addressing issues related to gene name variations, ultimately enhancing the efficiency and reliability of genetic research endeavors.

It is worth mentioning that all variations of gene names found in chordate and embryophyte genomes available in GenBank were used for standardizing nomenclature (available in GitHub). Therefore, SynGenes is limited to the data included up to 2023 (new variations may be included in new versions). Additionally, since we only utilized variations present in deposited genomes, variations found in gene sequences were not analyzed and thus not included. Similarly, SynGenes only includes data from coding and ribosomal genes. Therefore, if researchers intend to analyze tRNA genes, SynGenes is not recommended for such tasks.

In this way, SynGenes offers a solution for both standardizing gene nomenclatures, which can be implemented in different workflows through its Python class and providing a standardized search solution for specific markers in GenBank using its web form.

### Availability and requirements

Project name: SynGenes.

Project home page: https://github.com/luanrabelo/SynGenes

Operating system(s): Linux, Windows and MacOS.

Programming language: Python.

Other requirements: Python 3.10 + , Pandas > 2.30.0 and Requests > 2.0.1

License: MIT.

Any restrictions to use by non-academics: None.

All data and code for this work are available on the SynGenes GitHub repository (https://github.com/luanrabelo/SynGenes).

### Supplementary Information

**Additional file 1**: List of Chordata genomes analyzed in this study, including their taxonomic classification and accession numbers.

**Additional file 2**: List of Embryophyta genomes analyzed in this study, including their taxonomic classification and accession numbers

## Data Availability

SynGenes's source code and PyPI installation link are available at https://github.com/luanrabelo/SynGenes and https://pypi.org/project/SynGenes/, respectively. The list of genomes analyzed during the current study is available at the GitHub repository. The SynGenes website is available at https://luanrabelo.github.io/SynGenes/

## References

[1] Mullis K, Faloona F, Scharf S, Saiki R, Horn G, Erlich H (1986). Specific enzymatic amplification of DNA in vitro: the polymerase chain reaction. Cold Spring Harb Symp Quant Biol.

[2] Straiton J, Free T, Sawyer A, Martin J (2019). From Sanger sequencing to genome databases and beyond. Biotechniques.

[3] Artamonova VS, Kolmakova OV, Kirillova EA, Makhrov AA (2018). Phylogeny of salmonoid fishes (Salmonoidei) based on mtDNA COI gene sequences (barcoding). Contemp Probl Ecol.

[4] Liu Y, Johnson MG, Cox CJ, Medina R, Devos N, Vanderpoorten A (2019). Resolution of the ordinal phylogeny of mosses using targeted exons from organellar and nuclear genomes. Nat Commun.

[5] Li Q, Li L, Feng H, Tu W, Bao Z, Xiong C, et al. Characterization of the complete mitochondrial genome of basidiomycete yeast *Hannaella oryzae*: intron evolution, gene rearrangement, and its phylogeny. Front Microbiol. 2021;12. 10.3389/fmicb.2021.646567 PMC8193148 34122362

[6] Gulyaev S, Cai X-J, Guo F-Y, Kikuchi S, Applequist WL, Zhang Z-X (2022). The phylogeny of *Salix* revealed by whole genome re-sequencing suggests different sex-determination systems in major groups of the genus. Ann Bot.

[7] Irwin NAT, Tikhonenkov DV, Hehenberger E, Mylnikov AP, Burki F, Keeling PJ. Phylogenomics supports the monophyly of the Cercozoa. Mol Phylogenet Evol. 2019;130:416–23. 10.1016/j.ympev.2018.09.004 30318266

[8] Van Leeuwen P, Michaux J. Using eDNA for mammal inventories still needs naturalist expertise, a meta‐analysis. Ecol Evol. 2023;13. 10.1002/ece3.10788 PMC10701181 38077514

[9] Kumar A, Choudhury B, Dayanandan S, Latif M. Genetics and genomics tools in biodiversity conservation. 1st edition;2022.

[10] Magalhães M, Lyra ML, De Carvalho TR, Baldo D, Brusquetti F, Burella P, et al. Taxonomic review of South American butter frogs: Phylogeny, geographic patterns, and species delimitation in the *Leptodactylus latrans* Species Group (Anura: Leptodactylidae);2020.

[11] Lira NL, Tonello S, Lui RL, Traldi JB, Brandão H, Oliveira C (2023). Identifying fish eggs and larvae: from classic methodologies to DNA metabarcoding. Mol Biol Rep.

[12] Gibson TI, Carvalho G, Ellison A, Gargiulo E, Hatton-Ellis T, Lawson-Handley L (2023). Environmental DNA metabarcoding for fish diversity assessment in a macrotidal estuary: a comparison with established fish survey methods. Estuar Coast Shelf Sci.

[13] Landinez-Torres A, Panelli S, Picco AM, Comandatore F, Tosi S, Capelli E. A meta-barcoding analysis of soil mycobiota of the upper Andean Colombian agro-environment. Sci Rep. 2019;9:10085. 10.1038/s41598-019-46485-1 PMC6625999 31300737

[14] Fahmi MR, Kusrini E, Hayuningtiyas EP, Sinansari S, Gustiano R (2020). Dna barcoding using COI gene sequences of wild betta fighting fish from Indonesia: Phylogeny, status and diversity. Indones Fish Res J.

[15] Nakazato T. Survey of species covered by DNA barcoding data in BOLD and GenBank for integration of data for museomics. Biodiversity Information Science and Standards. 2020;4.

[16] Froufe E, Bolotov I, Aldridge DC, Bogan AE, Breton S, Gan HM (2020). Mesozoic mitogenome rearrangements and freshwater mussel (Bivalvia: Unionoidea) macroevolution. Heredity (Edinb).

[17] Chen C, Li Q, Fu R, Wang J, Deng G, Chen X (2021). Comparative mitochondrial genome analysis reveals intron dynamics and gene rearrangements in two *Trametes* species. Sci Rep.

[18] Bernt M, Merkle D, Ramsch K, Fritzsch G, Perseke M, Bernhard D (2007). CREx: inferring genomic rearrangements based on common intervals. Bioinformatics.

[19] Zhang J, Kan X, Miao G, Hu S, Sun Q, Tian W. qMGR: A new approach for quantifying mitochondrial genome rearrangement. Mitochondrion. 2020;52:20–3. 10.1016/j.mito.2020.02.004 32045715

[20] Zhang D, Gao F, Jakovlić I, Zou H, Zhang J, Li WX (2020). PhyloSuite: An integrated and scalable desktop platform for streamlined molecular sequence data management and evolutionary phylogenetics studies. Mol Ecol Resour.

[21] Chung M, Zhou J, Pang X, Tao Y, Zhang J. BioKDE: a deep learning powered search engine and biomedical knowledge discovery platform. In: BioCreative VII Challenge Evaluation Workshop, Virtual workshop. 2021. p. 254–9.

[22] Seal RL, Braschi B, Gray K, Jones TEM, Tweedie S, Haim-Vilmovsky L, et al. Genenames.org: the HGNC resources in 2023. Nucleic Acids Res. 2023;51:D1003–D1009. 10.1093/nar/gkac888 PMC9825485 36243972

[23] Bucklin A, Peijnenburg KTCA, Kosobokova KN, O’Brien TD, Blanco-Bercial L, Cornils A (2021). Toward a global reference database of COI barcodes for marine zooplankton. Mar Biol.

[24] Liu Y, Yao L, Ci Y, Cao X, Zhao M, Li Y (2021). Genetic differentiation of geographic populations of *Rattus tanezumi* based on the mitochondrial Cytb gene. PLoS ONE.

[25] Phadungsaksawasdi K, Sunantaraporn S, Seatamanoch N, Kongdachalert S, Phumee A, Kraivichian K (2021). Molecular analysis of mitochrondrial cytb of *Pediculus humanus capitis* in Thailand revealed potential historical connection with South Asia. PLoS ONE.

[26] Bernacki LE, Kilpatrick CW (2020). Structural variation of the turtle mitochondrial control region. J Mol Evol.

